# Living With Diabetes in Alberta: Patient and Caregiver Priorities for Diabetes Care, Management, and Treatment

**DOI:** 10.1111/hex.70587

**Published:** 2026-02-05

**Authors:** Sadia Ahmed, Paul Fairie, Iqmat Iyiola, Dorothy Nelson, Maria J. Santana

**Affiliations:** ^1^ Alberta Strategy for Patient Oriented Research, University of Calgary Calgary Alberta Canada; ^2^ Department of Community Health Sciences University of Calgary Calgary Alberta Canada; ^3^ Faculty of Agricultural, Life, and Environmental Sciences, University of Alberta Edmonton Alberta Canada; ^4^ Department of Pediatrics, Department of Community Health Sciences Cumming School of Medicine, University of Calgary Calgary Alberta Canada

**Keywords:** community consultation, diabetes care, patient engagement, patient partner, patient‐oriented research

## Abstract

**Introduction:**

Patients and caregivers living with diabetes experience multiple barriers to diabetes management. These include financial, geographic, and lack of culturally relevant diabetes education. Our aim was to understand the perspectives of patients living with diabetes on what should be prioritised in Alberta regarding diabetes care, management, and treatment. In this paper, we described our community engagement process and summarised the main priorities identified by Albertans regarding diabetes care, management, and treatment.

**Methods:**

Our team consisted of academic researchers, research & engagement staff, and two experienced patient partners, one with lived experience of Type 1 diabetes, and one family caregiver of Type 2 diabetes patients. We conducted a patient‐oriented qualitative study through focus groups with patients living with diabetes and caregivers across Alberta. Notes and reflections from the focus groups were qualitatively analyzed using content analysis in an inductive manner.

**Results:**

A total of 60 Albertans living with Type 1 (55%), Type 2 (42%), pre‐diabetes (2%), and latent autoimmune diabetes of adults (LADA) (3%) were engaged in focus group and interview sessions. The results of the 11 focus groups and two interviews with 60 Albertans were summarised into three main themes, and eight priorities (subthemes) identified within these themes: (1) **Access to resources** (medications and technology, social and mental health supports, physicians and specialists, and physical activity). (2) **Compassionate care**. (3) **Education & research** (awareness about diabetes & diabetes research, and improved patient and community informed research capacity).

**Conclusion:**

This work was undertaken to support the efforts of the Provincial Diabetes Working Group. Community consultations such as this are critical in informing health policy, as they ensure that decision‐making is grounded in the lived experiences, needs, and priorities of patients and caregivers. Through the highlighted barriers to care, gaps in education, and culturally specific needs, these consultations provide actionable evidence to guide policy development, programme planning, and resource allocation.

**Patient or Public Contribution:**

Patient partners were involved in this study. Our two patient partners supported the recruitment of participants, facilitated focus groups and semi‐structured interviews, and supported the interpretation of the results. This included the review of the main priorities identified. They also contributed to this manuscript as co‐authors.

## 1 Background

In 2024, approximately 5,805,000 Canadians (15%) were living with diabetes—(Type 1 and Type 2 diagnosed and undiagnosed) Type 2 diabetes accounting for 90%–95% of cases. In Alberta, an estimated 13% of the population live with diabetes either Type 1 or Type 2 diagnosed or undiagnosed, equivalent to 600,000 Albertans [1]. Type 2 diabetes symptoms often develop slowly, leading to many individuals unaware they are living with the condition and therefore being undiagnosed [2]. However, undiagnosed diabetes is reported to carry similar risk of mortality when compared to those diagnosed with diabetes [2].

We identified multiple barriers for people living with diabetes. Factors that influence one's ability to manage diabetes include educational level, acculturation, trust and respect for physicians, and social norms [3]. One Alberta‐based study highlighted that patients reported financial barriers related to self‐management supplies (e.g., glucometers and glucose testing strips), prescription medications, and access to healthy food [4]. Interviews conducted in 2018 with five members of First Nations communities in Alberta further revealed barriers such as transportation costs and logistical challenges, particularly due to the geographic isolation of many Indigenous communities [5]. Geographic isolation was also associated with limited access to fresh and nutritious food. In addition, a lack of culturally relevant diabetes education and the stigma surrounding diabetes were cited as barriers [5, 6, 7]. Another study that involved Indigenous patients across three Canadian provinces identified the colonial legacy of healthcare systems has contributed to treatment avoidance, mistrust of providers, inequitable care, and systemic power imbalances between patients and healthcare professionals [8]. A recent study in Alberta demonstrated that patients hospitalised due to diabetes reported lower patient experience scores than patients hospitalised because of other chronic conditions [9]. Older adults in the United States worried about access to diabetes supplies with changes in insurance plans [10]. Globally, financial burden and strict eligibility criteria by insurance providers led to disparities in the access to diabetes technology [11].

These findings underscore the importance of engaging people living with diabetes, particularly those from marginalised communities, in the development of health policies [12, 13]. Their lived experiences provide critical insights into systemic barriers and can help inform more equitable, patient‐centred approaches to diabetes prevention, management, and care delivery.

To address the alarming situation for Albertans living or at risk of developing diabetes, a provincial diabetes working group was established to review current diabetes care in Alberta and provide recommendations for the prevention, diagnosis, treatment, and management of diabetes to the Ministry of Health (now called the Ministry of Primary and Preventative Health Services) [14]. Membership included healthcare providers, representatives from non‐profit organisations such as Diabetes Canada, health services researchers, representatives from Indigenous communities, and patient partners (Albertans living with Type 1 or Type 2 diabetes, including those using insulin pumps). One of the co‐authors of this manuscript was a member of the working group.

One of the group's key action items was to strengthen the involvement of individuals living with diabetes and their family caregivers in identifying priorities for diabetes care, management, and treatment across the province. This meant ensuring people with lived experience were actively engaged in developing priorities. In response, our team which included patient partners, conducted consultations engaging patients and caregivers from across Alberta to inform the work of the diabetes working group.

Our aim was to understand the perspectives of patients living with diabetes on what should be prioritised in Alberta regarding diabetes care, management, and treatment through a patient‐oriented research approach. This paper described our community engagement process and summarised the main priorities identified by Albertans regarding diabetes care, management, and treatment.

## 2 Methods

### 2.1 Public Engagement Process

The Alberta Strategy for Patient‐Oriented Research (SPOR) SUPPORT Unit Patient Engagement Team supports researchers and patients to work together on the research that matters most to patients and regularly engages with Albertans across the province on projects that match their priorities [15].

In this study we used a patient‐oriented research approach to conduct this qualitative project.

**Figure 1:**
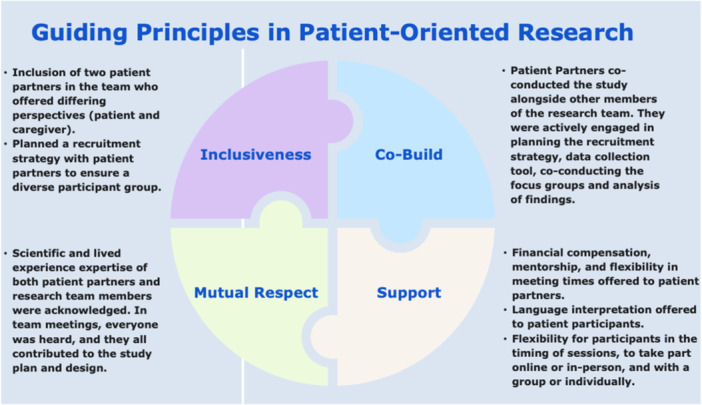
Patient engagement strategies used in this project aligning with the four guiding principles of patient engagement in patient‐oriented research.

To guide the development and implementation of this project, we adopted the Strategy for Patient‐Oriented Research (SPOR) Patient Engagement Framework [16, 17]. This framework is grounded in four guiding principles. First, *inclusiveness* ensures the integration of diverse patient perspectives throughout the research process. Second, *support* emphasises the provision of adequate resources and flexibility for patient partners, including training, financial compensation, and mentorship, as well as supports for patient participants such as language interpretation and the option to engage either online (e.g., via Zoom) or in person. Third, *mutual respect* underscores the importance of all team members—including researchers and patient partners—acknowledging and valuing both scientific expertise and lived experience. Finally, *co‐build* highlights the collaborative approach taken to jointly develop the community engagement strategy and process with active involvement of patient partners. Figure 1 maps our strategies to the four guiding principles.

Our team consisted of academic researchers, research & engagement staff, and two experienced patient partners, one with lived experience of Type 1 diabetes, and one family caregiver of Type 2 diabetes patients. Both patient partners also were graduates of the Patient and Community Engagement Research (PaCER) programme [18], therefore they had training experience in qualitative community‐based research. The two patient partners brought their lived experiences and qualitative research experiences to this study. Their previous health research experience and training prepared them in co‐conducting the community consultations.

With our two patient partners, we designed the engagement process, and recruitment materials including the recruitment poster. Our patient partners supported the recruitment of participants, facilitated focus groups and semi‐structured interviews, and supported the interpretation of the results including reviewing the main priorities identified. The focus group/interview guide was reviewed by all research team members. The questions in the guide asked participants about their overall experiences living with diabetes, what their priorities were on imp roving diabetes prevention (for Type 2), priorities for improving diabetes care for adults and children, priorities for diabetes treatment, and priorities regarding research and development about diabetes care. The study has been approved by the University of Calgary Conjoint Health Research Ethics Board (REB23‐1422).

### 2.2 Recruitment Process

Sampling was purposive, as we sought participants with lived experience of diabetes in Alberta, or caregivers. The recruitment criteria were as follows: living in Alberta, age 18 years or older, and living with diabetes or a caregiver of someone living with diabetes. The recruitment poster was shared with different community and partner organisations across Alberta to identify Albertans with lived experience of diabetes who were willing to share their insights. See Table 1 for a list of organisations.

**Table 1:** Alberta organisations supporting the recruitment.

Calgary Zone	Calgary Catholic Immigration Society ActionDignity Ukrainian Youth Association Punjabi Community Health Services Calgary Multicultural Seniors Association Women's Centre Calgary Alberta Network of Immigrant Women Youth Central Trellis Society Gateway Mosaic Clinic
Edmonton Zone	Diabetes Youth Network Edmonton
North Zone	Grande Prairie Centre for Newcomers Family and Community Support Services (FCSS) Grande Prairie Family and Community Support Services (FCSS) Wood Buffalo Region Family and Community Support Services (FCSS) Cold Lake
South Zone	L'Association Francophone de Brooks Primary Care Network Chinook (Lethbridge) South Peace News Family and Community Support Services (FCSS) Lethbridge
Provincial	Diabetes, Obesity and Nutrition Strategic Clinical Network (SCN) Foundation for the Voice of Immigrants in Canada for Empowerment (VOICE) Alberta International Medical Graduates Association (AIMGA) Local Immigrant Partnership Network Imagine Citizens Network Rural Development Network Albertans4HealthResearch Collaborative Council Alberta Youth Health Advisory

We also shared the recruitment poster with our patient partner provincial network, Albertans4HealthResearch [15], as well as with current students and alumni of the PaCER programme [18]. By leveraging these networks, patient partners further disseminated the poster through relevant Facebook groups, community organisations, diabetes‐focused groups, personal and professional networks, diabetes clinics, and pharmacies across the province. This broad outreach was intended to maximise awareness of the opportunity and ensure the inclusion of diverse perspectives. Anyone interested in participating, filled out an online form which asked for contact details, what their experience with diabetes was (e.g., Type 1 patient, Type 2 patient, or other type, or whether they were a family caregiver), and whether interpretation would be required. The online form allowed the research team to screen participants and identify if they were eligible for the study.

### 2.3 Data Collection and Analysis

We conducted eleven focus groups, and two semi‐structured interviews with Albertans living with diabetes and caregivers between December 2023‐February 2024. Albertans from urban, rural, and remote geographical areas were engaged in these consultations. All focus groups and interviews were conducted online over Zoom by two patient partners and/or a health services researcher (SA, MSc degree in Health Services Research), who all identified as women and co‐authors of this paper. The focus groups were audio‐recorded, notes were taken during the focus group to summarise main priorities and reflections were written afterwards by the facilitators. Focus groups and interviews were scheduled for 90 min, and each focus group had around 4–8 participants. One interview was conducted with the support of a Spanish interpreter, and one focus group was conducted with the support of a Farsi interpreter.

Notes and reflections from the focus groups were qualitatively analyzed using content analysis in an inductive manner [19]. This analysis involved the systematic coding and interpretation of textual data to identify patterns, categories, and themes. The process began with familiarisation, where the health services researcher read through the data to gain an overall sense of the content. Then, segments of text were coded based on their meaning and relevance to the research question. The codes were developed during the coding process from the data itself, rather than following a pre‐determined codebook. The main codes derived from the research question were priorities for diabetes management, treatment, and care. These codes were then grouped into categories that captured recurring ideas, which were further refined into broader themes. Throughout the process, the health services researcher iteratively compared data segments, codes, and categories to ensure consistency and validity. This involved re‐reading through the notes takes from the focus group, and reflections from the facilitators on the main priorities identified by the participants. A coding tree of our analysis was attached as an appendix. We employed strategies to increase dependability, we kept an audit trail of all notes and reflections, and complete record of study decisions. The final step involved interpreting the themes in relation to the study objectives and we situated the findings within the broader literature. To enhance the credibility of our findings, the main themes (priorities) identified were shared back with the research team for further discussion and refinement through the process of peer debriefing. The peer debriefing process validated the coding and interpretation process. The finalised themes (priorities) described below in this manuscript.

In the online interest form, 125 individuals expressed interest in participating in a focus group. Of those 125, 12 had confirmed their interest, but did not show up for the online focus groups. The other individuals either were no longer available or were hard to get a hold of. After conducting focus groups with 60 individuals, we chose to not conduct any more focus groups. This is because we had identified similar priorities/themes across the focus groups and had not identified new priorities/themes. Additionally, the timeframe of the study also limited how many focus groups we held. After speaking with 60 Albertans, and reviewing the data, we have identified a comprehensive list of priorities from the perspectives of patients and caregivers. Our data collection was focused on identifying meaningful insights from our participants. Rather than achieving thematic saturation, the process of identifying priorities from Albertans was sufficiently well developed in the context of our research question [20, 21].

## 3 Results

A total of 60 Albertans living with Type 1 (55%), Type 2 (42%), pre‐diabetes (2%), and latent autoimmune diabetes of adults (LADA) (3%) were engaged in focus group sessions. More than half (55%) of participants were from the Calgary area, 22% were from Edmonton, 10% were from the North Zone, 10% were from Central Zone, and 3% were from South Zone. Majority of participants were women (77%), and majority were of European background (69%). Age range of participants varied from 18 to 79 years. A summary of the demographics presented below in Table 2.

**Table 2:** Summary of demographics.

	**Total *n* (%)**
**Place of residence in AB**	
Calgary	33 (55)
Edmonton	13 (22)
North Zone (e.g., Grand Prairie, Fort McMurray)	6 (10)
Central Zone (e.g., Red Deer)	6 (10)
South Zone (e.g., Lethbridge, Medicine Hat)	2 (3)
**Gender**	
Man	13 (22)
Woman	46 (77)
Prefer not to answer	1 (2)
**Cultural background**	
African	3 (5)
East Asian	1 (2)
European	40 (69)
Hispanic or Latinx	2 (3)
First Nations or Indigenous	3 (5)
South Asian	1 (2)
Southeast Asian	4 (7)
Prefer not to answer	4 (7)
**Age range (years)**	
18–24	1 (2)
25–34	8 (13)
35–44	9 (15)
45–54	21 (35)
55–64	9 (15)
65–79	11 (18)
Prefer not to answer	1 (2)
**Type of diabetes you/your loved one diagnosed with**	
Type 1	33 (55)
Type 2	25 (42)
Pre‐diabetes	1 (2)
LADA	2 (3)

### 3.1 Summary of Main Priorities

Patients and caregivers were appreciative of having the opportunity to share their experience and priorities on diabetes care, treatment, and prevention in Alberta. Some participants also commented and appreciated meeting other patients and caregivers in the focus groups and learning from each other. Most patients and caregivers expressed the stress of trying to manage and cope with their diabetes. Living with diabetes was described as a full‐time job, difficult, and frustrating. Some described diabetes as a solitary disease. During the focus groups, patients and caregivers shared ways they managed their diabetes, resources, and supports they currently had or wish they had, and priorities Alberta can focus on.

The results of the 11 focus groups and two interviews with 60 Albertans can be broadly themed into three groups (themes):

1. **Access to resources** (medications and technology, social and mental health supports, physicians and specialists, and physical activity)
2. **Compassionate care**
3. **Education & research** (awareness about diabetes & diabetes research, and improved patient and community informed research capacity).

**Figure 2:**
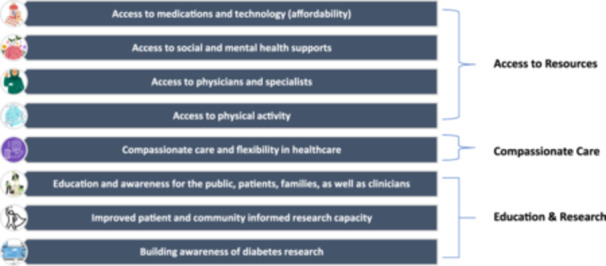
Eight priorities identified by patients and caregivers in Alberta grouped by the three themes.

The eight priorities (sub‐themes) identified are discussed in detail in this section, and illustrative quotes from the focus groups and interviews are included. See Figure 2 and Supporting Information S1: Appendix for details.

## 4 Theme: Access to Resources

In this theme we described four priorities of patients and caregivers relating to access to resources such as access to medications and technology, access to social and mental health supports, access to physicians and specialists, and access to physical activity.

### 4.1 Priority 1—Access to Medications and Technology (Affordability)

Albertans spoke about the importance of having access to funding for medications and technology for diabetes. For instance, expanding the coverage of continuous glucose monitors (CGMs), insulin, and glucagon pen, and other medications. Some patients mentioned they were on the Insulin Pump programme, and they described the programme greatly improved their quality of life while living with Type 1 diabetes. Expanding coverage and access to other technology was described as crucial for many of the participants. For instance, one patient said:

I'm on the Insulin Pump Program that the government does pay for. But they threatened to take us off that and quit the program, which scares me because that would greatly change my quality of care I could receive or afford to receive. And I'd like to see them covering things like sensors, not only for Type 1 diabetics, but for Type 2 too, because like that will… like why wait until people are on insulin and are bad enough off that they need – their quality of life has gone downhill. Why not prevent them problems by giving them access to the things they need to prevent their disease from hindering their life?

(T1D Patient 2)

Patients provided different ways they managed their diabetes and wanted other patients to have access to the same technology.

If every person who was a diabetic, regardless of Type 1 or Type 2 had access to…sensors that will send a reading to your phone, or your insulin pump, or whatever system you happen to be using, every 5 minutes – my thing sends a report to my insulin pump. And I know my blood sugar every moment of the day, and that's been life changing for me. Testing your blood three times a day is not, or five times a day even, isn't an effective way to manage diabetes. So if they could make it less expensive, or support people some way, to be able to have access to those, because it's quite cost prohibitive for I think a lot of people.

(T1D Patient 2)

Some participants also mentioned the affordability of food being an issue for them in managing their diabetes.

It would probably much likely improve my diet, more solid, more diabetic type of menus, rather than just kind of – because I kind of buy stuff that's on sale as I can kind of afford it. So affordability is a big one for me.

(T2D Patient 1)

### 4.2 Priority 2—Access to Social and Mental Health Supports

Diabetes was mentioned by many of the participants as a stressful disease. Many participants mentioned the need for Alberta to prioritise social and mental health supports for patients and their families. Some examples given were mental health services, and support groups especially for children and seniors.

One family caregiver spoke about the isolation her mom faced:

Seniors support group. Yes, so because seniors, like my mom, I believe…she needs to talk to someone to share experiences. And she has only me to tell everything. And I may not feel the same way, I may not be compassionate enough all the time. But I believe if there will be a group of, there will be classes like that, or community support, groups like that, for seniors, together with a diabetes educator, who can have some sessions with them, and they can socialize, that would be definitely something that mom – I would take my mom there. And it will improve her psychological state related…to diabetes and side effects.

(T2D Caregiver 2)

Another caregiver commented on newcomer populations being underserved:

If you think about newcomers and people who are priority populations, it's people that might not have access to diabetes care and prevention. And I think that we can't forget about those populations as well, because we're just going to overburden the system so that nobody is going to get the care that they deserve, because there's just going to be so much diabetes around us that we have to contend with… So I think they need to not forget about those priority populations.

(T2D Caregiver 3)

Some patients mentioned the transition for youth with Type 1 diabetes when they transition from pediatric to adult care to be a difficult and isolating period, and therefore, having access to support to make that transition smooth was needed.

### 4.3 Priority 3—Access to Physicians and Specialists

Many participants commented on there not being enough diabetes specialists such as nurse educators, dieticians, and endocrinologists in Alberta. Some participants lived outside of major cities such as rural towns and didn't have access to any diabetes specialists in their region. Some participants also mentioned that access to nurse educators in schools would support children who live with diabetes.

One patient commented on access to specialist care in Red Deer, Central Zone:

One thing that's lacking here in Central Zone is we don't have an endocrinologist in Red Deer. We don't have one at the hospital, we don't have one at the diabetic education centre. They consult with Edmonton and Calgary, but there isn't one here. So that is a huge hole in the system for Red Deer in Central Zone. So bringing those specialties into place, I think would really help to improve the care for adults living with diabetes and improve the peace of mind of the care partners for those people as well.

(T2D Patient 5)

Another patient mentioned access to primary care for patients should be improved:

Ways our government can improve is improved access to primary care, just improving people's access to a family physician, to a nurse practitioner or to a diabetes educator.

(T2D Patient 8)

### 4.4 Priority 4—Access to Physical Activity

To support prevention efforts for Type 2 diabetes, some participants commented that patients need to have access to affordable spaces to engage in physical activity.

One patient commented:

I would say improving people's access to exercise, people's access to getting more active and lowering the cost of those things. So getting access to getting into the pool for people that have low mobility, getting access to indoor walking tracks, especially for seniors with lower incomes, would really make a big difference.

(T2D Patient 15)

## 5 Theme: Compassionate Care

This theme described compassionate care and flexibility in healthcare, specifically from the healthcare system.

### 5.1 Priority 5—Compassionate Care and Flexibility in Healthcare

Some participants spoke about not feeling listened to and taken seriously when they tried to express their concerns about their diabetes management with their physicians. Participants expressed the need for their care teams to be open‐minded, compassionate, and listen to the needs of patients.

For example, one patient explained that healthcare professionals should be flexible in the rules or guidelines for diabetes management:

Everyone's body is different so they give you all these blanket rules like – you know in terms of like treating your lows and like how long you have to wait and everything like that. But I found that there is a bit of a need for flexibility because everyone's body reacts differently. I kind of tried to discuss that with them and say like, “Hey, actually, this isn't working for me so I'm doing this” and then they were like, “No, no, no, you have to do this.

(T1D Patient 6)

## 6 Theme: Education and Research

The theme education and research related to three priorities of patients and caregivers. This included education and awareness for the public, patients, families, as well as clinicians, improved patient and community informed research capacity, and lastly, building awareness of diabetes research.

### 6.1 Priority 6—Education and Awareness for the Public, Patients, Families, as Well as Clinicians

When discussing prevention initiatives, or addressing stigma associated with diabetes, many patients and caregivers advocated for educating the public and healthcare professionals. Some participants mentioned the need for diabetes awareness in schools.

One patient commented:

Education on all parts for the people living with the disease. I learn something new every single day that I didn't know. I've only had Type 1 for three years but every day I learn something new; every single day. And again the general public are unaware…or re‐educating the public because I still think that there is that stigma that it's you know people that are overweight and sedentary that get this disease and that's not the case.

(T1D Patient 1)

Caregivers sought more information on prevention for Type 2 diabetes and risks associated. For instance, one caregiver said:

I'd like to have more prevention initiatives in place. For example, I'm wondering if there are things that I'm doing that are putting me at risk (I don't have diabetes‐‐ I'm 54). And for my children‐‐ they are 13, 11, 10 years old… how can I make sure their risk is decreased?

(T2D Caregiver 2)

Some patients also wanted healthcare professionals to be educated about diabetes and the different treatments and technologies associated with diabetes management. One patient noted:

And I think in healthcare, we have to realise that… everybody's accountable, it's spread across a complete spectrum of health care, whether it be primary care or acute care. But I think we're responsible for our own health, but I also think that the system is responsible to ensure that we are educated about our health. And that's why I say accountability, it falls within primary care, but it also falls within the acute care side of it.

(T2D Patient 3)

### 6.2 Priority 7

#### 6.2.1 Improved Patient and Community Informed Research Capacity

Some participants brought up the need for opportunities for patients and caregivers to share their experiences and thoughts on diabetes. Participants commented on the need for ongoing and continued engagement of patient and families in care and in research. Participants also mentioned the need to be engaged in their care by being involved in shared decision making with clinicians.

Some participants mentioned that community members need to involved in setting priorities and identifying needs.

One caregiver commented:

Connecting academics to community members, those who are living with diabetes, and the people that care for them. So I think doing more community based research, so what's needed and what's working, where are the gaps. And I think that maybe some of the gaps – [University of Alberta Researcher] has done a lot of work around like First Nations communities, there's increasing prevalence of diabetes in that population.

(T2D Caregiver 17)

### 6.3 Priority 8—Building Awareness of Diabetes Research

Most participants were not aware of current research for diabetes in Canada and internationally. Patients and caregivers commented that they would like to know about current research. Some participants were aware of diabetes research and mentioned the need for continued funding for diabetes research.

One patient said:

I think that the Edmonton Protocol is great that people are willing to give their money to diabetes and make donations, but this is ridiculous. This should be funded – and funded substantially. Because we, as Type 1 s and Type 2 s, if we do not manage our blood sugars, we're going to cost the system a whole lot of money. And we are going to suffer, and our families are going to suffer, and our children are going to suffer, and it's not acceptable.

(T1D Patient 26)

## 7 Discussion

These eight priorities outline the recommendations provided by Albertans on diabetes care in Alberta. This community engagement process allowed us to recruit participants from across Alberta to explore their experiences and priorities regarding diabetes care. This study provided evidence for the Provincial Diabetes Working Group for their report to the Ministry of Health (now called the Ministry of Primary and Preventative Health Services). The themes identified in our study align with findings from other research. For example, affordability of medications, supplies, and food emerged as a key priority for diabetes management, consistent with previous studies [4, 5, 22]. Education and awareness of diabetes were also highlighted as important by both patients and caregivers, paralleling findings in other diabetes research [5, 23]. Our priorities align with national diabetes organisations, such as Diabetes Canada which advocated for the priorities of access and affordability of care, access to health services which includes food security, timely access to diabetes medications, and accommodating individuals at their places of employment and in schools. Diabetes Canada has also advocated for the engagement of patient and community partners in health policy and programme development. The American Diabetes Association as well align with our findings, as they highlighted the following priorities of the diabetes community: access to adequate and affordable health care, funding support for diabetes research, and equitable treatment of people living with diabetes to access educational and employment opportunities [24]. Our study did not delve into possible sex and gender differences in barriers and priorities identified by those living with diabetes. However, exploring those differences may provide additional insight into how certain barriers and priorities impact different genders, and therefore different strategies needed to attend to those priorities. For instance, Logan et al. [25] found response differences among men and women on whether their diabetes care team supported their physical activity. Even though both men and women confirmed their healthcare providers did not provide specific strategies for staying active, women reported they felt supported by their healthcare providers while men did not [25]. A study conducted in rural Nebraska identified lack of access to specialised care, importance of technology for managing Type 1 diabetes, and importance of bringing awareness about diabetes to others [26]. Our study also identified those as key priorities; however, it wasn't specific to the rural communities, but also larger cities like Edmonton and Calgary. Additionally, even with differences in healthcare systems, access to healthcare services was identified as a barrier and priority between our studies and other US based studies.

The addition of our patient‐oriented approach, that engaged Albertans, was a notable strength of our study. Specifically, the inclusion of the two patient partners in this study, a patient and caregiver, throughout all stages of the project, including study design, recruitment strategy development, creation of recruitment materials, data collection and analysis, and manuscript preparation. The integration of our patient and caregiver partners enabled the co‐development of the recruitment strategy, ensuring representation across Alberta. Our partners leveraged their networks, including Facebook and community groups, to promote the study. They also contributed to the framing of focus group questions in a way that was meaningful to participants. During the focus groups, they facilitated discussions, probed patient priorities, and provided reflections that informed data interpretation. Across western countries, patient partners have been actively engaged on research projects. In Canada, studies such as by Watson et al. [27] describe the engagement of patient partners in data collection, and another study involved two patient advisors from the Patient Engagement Team at The Ottawa Hospital in designing a patient survey [28]. Davichi and Ferrari [29] collaborated with homeless and key community stakeholders to develop a diabetes prevention and management support initiative for the homeless population. The Patient‐Centered Outcomes Research Institute provided funding support to US Clinical research and emphasised the engagement of patients and caregivers in all aspects of PCORI‐funded research. Within their funding database, 234 completed diabetes research studies were identified that engaged patients, families, and community members [30].

Another strength of this study was the breadth of our community engagement process, which allowed us to recruit participants from diverse geographic zones in Alberta, varied age groups, different types of diabetes, and multiple cultural backgrounds, with 33% of participants identifying as non‐European. Engaging participants from diverse backgrounds is essential in public consultations to ensure comprehensive representation [31]. Additionally, including the caregiver perspective allowed for additional insights to diabetes care, management, and treatment. Including caregivers in research and diabetes education is necessary for diabetes care. In a 2023 systematic review, caregiver support and involvement in Type 2 diabetes education has been shown to improve patient outcomes, such as reduced body mass index and HbA1c, enhanced knowledge, and self‐efficacy [32]. We were also well equipped within our team to provide interpretation in multiple languages, including Tagalog, French, Hindi, Bengali, through the diverse languages spoken in our team as well through our connections with partner organisations that provide support in multiple languages.

A limitation of our study was that all our focus groups were conducted online. We may have missed the perspectives of Albertans with limited access to computers or insufficient digital literacy to participate in virtual sessions. The recruitment timeline also impacted our ability to recruit and engage participants from different backgrounds. We could not recruit many participants from the 18–24 age group, as well as those from South and East Asian backgrounds. Data analysis was led by one research team member due to time constraints. Ideally, it is recommended to have multiple coders in inductive content analysis to develop a richer and more nuanced interpretation of the data [33]. However, to address this, we shared the findings with supporting quotes after the initial coding process with the rest of the team for further feedback and discussion on the interpretation. This process of peer debriefing strengthened the credibility of our research.

This work was undertaken to support the efforts of the Provincial Diabetes Working Group. Community consultations such as this are critical in informing health policy, as they ensure that decision‐making is grounded in the lived experiences, needs, and priorities of patients and caregivers. By highlighting barriers to care, gaps in education, and culturally specific needs, these consultations provide actionable evidence to guide policy development, programme planning, and resource allocation. These findings were reported back to the Provincial Diabetes Working Group and integrated in their recommendations to the Ministry of Health (now called the Ministry of Primary and Preventative Health Services). Our study has provided a blueprint on how to engage patients, families, and communities through a patient‐oriented research approach. Engaging patients and caregivers throughout the research process strengthened the relevance and validity of our findings, supported the design of patient‐centred and equitable policies, and fostered trust and ongoing collaboration between healthcare systems, policymakers, and communities. Future directions include identifying priorities for further research in diabetes care, management, and treatment.

## Author Contributions

**Sadia Ahmed, Paul Fairie, Iqmat Iyiola, Dorothy Nelson** and **Maria J. Santana:** conceptualisation, investigation, methodology, writing – original draft, writing – review and editing, visualisation, formal analysis, project administration, data curation.

## Ethics Statement

The study has been approved by the University of Calgary Conjoint Health Research Ethics Board (REB23‐1422).

## Consent

Participants provided informed consent to participate in this study.

## Conflicts of Interest

The authors declare no conflicts of interest.

## Supporting information

Appendix.

coding tree.

## Data Availability

All data generated or analyzed during this study are included in this published article [and its Supporting Information files].

## References

[1] Diabetes Canada. Diabetes in Canada Estimated Prevalence and Cost of Diabetes diabetes.ca: Diabetes Canada; 2025.

[2] M. Evans , A. R. Morgan , D. Patel , et al., “Risk Prediction of the Diabetes Missing Million: Identifying Individuals at High Risk of Diabetes and Related Complications,” Diabetes Therapy 12, no. 1 (2021): 87–105. 33190216 10.1007/s13300-020-00963-2 PMC7843706

[3] C. Tørris and L. Nortvedt , “Health Literacy and Self‐Care Among Adult Immigrants With Type 2 Diabetes: A Scoping Review,” BMC Public Health 24, no. 1 (2024): 3248. 39578821 10.1186/s12889-024-20749-6 PMC11583541

[4] D. J. T. Campbell , B. J. Manns , B. R. Hemmelgarn , C. Sanmartin , A. Edwards , and K. King‐Shier , “Understanding Financial Barriers to Care in Patients With Diabetes: An Exploratory Qualitative Study,” Diabetes Educator 43, no. 1 (2017): 78–86. 27920081 10.1177/0145721716679276

[5] S. Kulhawy‐Wibe , K. M. King‐Shier , C. Barnabe , B. J. Manns , B. R. Hemmelgarn , and D. Campbell , “Exploring Structural Barriers to Diabetes Self‐Management in Alberta First Nations Communities,” Diabetology & Metabolic Syndrome 10, no. 1 (2018): 87. 30524507 10.1186/s13098-018-0385-7 PMC6276258

[6] M. C. Tremblay , M. Bradette‐Laplante , H. O. Witteman , et al., “Providing Culturally Safe Care to Indigenous People Living With Diabetes: Identifying Barriers and Enablers From Different Perspectives,” Health Expectations 24, no. 2 (2021): 296–306. 33350572 10.1111/hex.13168 PMC8077144

[7] J. Jordan , J. A. Manski‐Nankervis , M. Read , T. Skinner , J. Speight , and E. Holmes‐Truscott , “The Seagull Theory: Where People Fly in, Gather Information Fly out and Nothing Ever Comes About’: A Qualitative Exploration of Barriers and Enablers to Research Participation Among Adults With Type 2 Diabetes Living in Australian Rural Communities,” Diabetic Medicine 42, no. 5 (2025): e70027. 40122592 10.1111/dme.70027 PMC12006560

[8] K. M. Jacklin , R. I. Henderson , M. E. Green , L. M. Walker , B. Calam , and L. J. Crowshoe , “Health Care Experiences of Indigenous People Living With Type 2 Diabetes in Canada,” Canadian Medical Association Journal 189, no. 3 (2017): E106–E112. 28246155 10.1503/cmaj.161098 PMC5250516

[9] K. A. Kemp , P. Fairie , and M. J. Santana , “Patient Experiences With Hospitalization Due to Diabetes in Alberta, Canada: A Cohort Study Using Survey and Administrative Data,” Canadian Journal of Diabetes 48, no. 8 (2024): 544–550. 39424274 10.1016/j.jcjd.2024.10.005

[10] A. S. Hughes , K. Uddin , M. Krupar , E. VanDyke , and H. L. Stuckey‐Peyrot , “They Did Not Anticipate Anybody Living a Full Life”: Perspectives on Aging With Type 1 Diabetes,” Diabetes Spectrum 38 (2025): 259–265. 40823600 10.2337/ds24-0045 PMC12357191

[11] R. B. Conway , J. Snell‐Bergeon , K. Honda‐Kohmo , et al., “Disparities in Diabetes Technology Uptake in Youth and Young Adults With Type 1 Diabetes: A Global Perspective,” Journal of the Endocrine Society 9, no. 1 (2025): bvae210. 10.1210/jendso/bvae210 PMC11655873 39703363

[12] M. S. Martinez‐Cruz , N. Namasingh , A.‐S. Alexopoulos , B. C. Batch , M. J. Crowley , and H. B. Bosworth The Forgotten‐Overcoming Challenges in Diabetes Care for Marginalized Populations. Expert Review of Endocrinology & Metabolism. 2025(just‐accepted). 10.1080/17446651.2025.2526200 PMC13261768 40600803

[13] F. Hill‐Briggs , N. E. Adler , S. A. Berkowitz , et al., “Social Determinants of Health and Diabetes: A Scientific Review,” Diabetes Care 44, no. 1 (2020): 258–279. 33139407 10.2337/dci20-0053 PMC7783927

[14] Government of Alberta . Diabetes Working Group Government of Alberta2023 [2025 Oct 2]. https://www.alberta.ca/diabetes-working-group.

[15] Alberta Strategy for Patient Oriented Research . Albertans 4 Health Research AbSPORU Patient Engagement Team; 2025 [2025 September 17]. https://app.betterimpact.com/PublicOrganization/25809ea0-7311-40db-bfac-aaa0e28ba518/1.

[16] Canadian Institutes of Health Research . Strategy for Patient‐Oriented Research ‐ Patient Engagement Framework 2019 [2025 March 6]. https://www.cihr-irsc.gc.ca/e/48413.html.

[17] M. J. Santana , D. Duquette , P. Fairie , et al., “Patient‐Identified Priorities for Successful Partnerships in Patient‐Oriented Research,” Research Involvement and Engagement 8, no. 1 (2022): 49. 36071538 10.1186/s40900-022-00384-4 PMC9450417

[18] University of Calgary . Patient and Community Engagement in Research University of Calgary; 2025 [2025 September 17]. https://www.ucalgary.ca/patient-community-engagement-research.

[19] A. J. Kleinheksel , N. Rockich‐Winston , H. Tawfik , and T. R. Wyatt , “Demystifying Content Analysis,” American Journal of Pharmaceutical Education 84, no. 1 (2020): 7113. 32292185 10.5688/ajpe7113 PMC7055418

[20] M. Tight , “Saturation: An Overworked and Misunderstood Concept?,” Qualitative Inquiry 30, no. 7 (2024): 577–583.

[21] V. Braun and V. Clarke , “To Saturate or Not to Saturate? Questioning Data Saturation as a Useful Concept for Thematic Analysis and Sample‐Size Rationales,” Qualitative Research in Sport, Exercise and Health 13, no. 2 (2021): 201–216.

[22] F. M. Siad , X. Y. Fang , M. J. Santana , S. Butalia , M. A. Hebert , and D. M. Rabi , “Understanding the Experiences of East African Immigrant Women With Gestational Diabetes Mellitus,” Canadian Journal of Diabetes 42, no. 6 (2018): 632–638. 29914780 10.1016/j.jcjd.2018.01.013

[23] S. C. Catapan , C. Vasconcelos Silva , D. Bird , et al., “Working Together to Improve Type 2 Diabetes Care: A Participatory Design Project to Address Identified Needs of People With Diabetes and Their Health‐Care Professionals,” Canadian Journal of Diabetes 48, no. 4 (2024): 250–258.e2.e2. 38365115 10.1016/j.jcjd.2024.02.001

[24] American Diabetes Association . Advocacy Priorities American Diabetes Association2025 [2025 Nov 21]. https://diabetes.org/advocacy/initiatives.

[25] J. E. Logan , M. Prévost , A.‐S. Brazeau , et al., “The Impact of Gender on Physical Activity Preferences and Barriers in Adults With Type 1 Diabetes: A Qualitative Study,” Canadian Journal of Diabetes 48, no. 6 (2024): 401–408. 38825148 10.1016/j.jcjd.2024.05.003

[26] V. D. Jewell , A. C. Wise , E. L. Knezevich , A. A. Abbott , B. Feiten , and K. Dostal , “Type 1 Diabetes Management and Health Care Experiences Across Rural Nebraska,” Journal of Pediatric Health Care 37, no. 1 (2023): 48–55. 36064764 10.1016/j.pedhc.2022.07.005

[27] K. E. Watson , K. Dhaliwal , E. Benterud , et al., “Managing Medications During ‘Sick Days’ in Patients With Diabetes, Kidney, and Cardiovascular Conditions: A Theory‐Informed Approach to Intervention Design and Implementation,” Canadian Journal of Diabetes 48, no. 4 (2024): 259–268.e4.e4. 38395301 10.1016/j.jcjd.2024.02.003

[28] P. Beamish , K. McNeill , A. Arnaout , and J. Malcolm , “Patient Perspectives on Virtual Care for Diabetes Management in the Era of COVID‐19,” Canadian Journal of Diabetes 47, no. 8 (2023): 636–642. 37437840 10.1016/j.jcjd.2023.07.001

[29] S. Davachi and I. Ferrari , “Homelessness and Diabetes: Reducing Disparities in Diabetes Care Through Innovations and Partnerships,” Canadian Journal of Diabetes 36, no. 2 (2012): 75–82.

[30] Patient‐Centered Outcomes Research Institute . Explore our Portfolio PCORI2025 [2025 Nov 21]. https://www.pcori.org/explore-our-portfolio?&keyword=diabetes&f[0]=status:3055.

[31] S. Mitchell , A. Bragg , I. Moldovan , et al., “Stigma as a Barrier to Participant Recruitment of Minority Populations in Diabetes Research: Development of a Community‐Centered Recruitment Approach,” JMIR Diabetes 6, no. 2 (2021): e26965. 33938811 10.2196/26965 PMC8129881

[32] J. Kim , J. Song , A. Tark , S. Park , and K. Woo , “Do Caregivers’ Involvement in Type 2 Diabetes Education Affect Patients’ Health Outcomes?: A Systematic Review and Meta‐Analysis,” Journal of Health Sciences 13, no. 3 (2023): 133–147.

[33] F. Coulston , F. Lynch , and D. F. Vears , “Collaborative Coding in Inductive Content Analysis: Why, When, and How to Do It,” Journal of Genetic Counseling 34, no. 3 (2025): e70030. 40305144 10.1002/jgc4.70030 PMC12042989

