# Supplementary material for: Living With Diabetes in Alberta: Patient and Caregiver Priorities for Diabetes Care, Management, and Treatment
Source: Health Expect. 2026 Feb 5;29(1):e70587. doi: 10.1111/hex.70587 (PMC12877417; doi:10.1111/hex.70587)
Supplement: Supplementary file 1 — Appendix. [file HEX-29-e70587-s001.docx]

**Appendix**

This appendix includes additional supporting quotes for each of the themes identified by Albertans as part of this process.

| **Priority** | **Summary** | **Supporting Quotes** |
| --- | --- | --- |
| **Access to medications and technology (affordability)** | Albertans spoke about the importance of having access to funding for medications and technology for diabetes. For instance, expanding the coverage of continuous glucose monitors (CGMs), insulin, and glucagon pen, and other medications.  Some participants also mentioned the affordability of food being an issue for them in managing their diabetes | “The need for funding is important…I’m concerned that when we do finally move towards funding CGMs better, that they’re going to drop the test strips. Please don’t. Some of us still rely very heavily on test strips, even on CGM” (**T1D Patient 19**)  “I would love to see CGMs covered just like they do in British Columbia and other provinces, as well as enhancing the insulin pump therapy program.” (**T1D Patient 22**)  “Affordability. Yeah, that's a big one, for everything, medication, prescriptions, type of thing and food. Proper food.” (**T2D Patient 14**)  “It would probably much likely improve my diet, more solid, more diabetic type of menus, rather than just kind of – because I kind of buy stuff that's on sale as I can kind of afford it. So affordability is a big one for me.” (**T2D Patient 1**)  “What happens when we don’t have insurance anymore because we are no longer employed and we can’t continue to purchase that insurance? So for me, for me personally, that’s a big concern for me. How do I pay for insulin? How do I how do I get access to the newest stuff – like the newest diabetes technology. And if the province isn’t going to cover – like how – I don’t know about any of you, but this is a definite concern of mine – is retirement savings and how I’m going to pay for my medication without insurance.” (**T1D Patient 10**)  “I’m on the Insulin Pump Program that the government does pay for. But they threatened to take us off of that and quit the program, which scares me because that would greatly change my quality of care I could receive or afford to receive. And I’d like to see them covering things like sensors, not only for Type 1 diabetics, but for Type 2 too, because like that will… like why wait until people are on insulin and are bad enough off that they need – their quality of life has gone downhill. Why not prevent them problems by giving them access to the things they need to prevent their disease from hindering their life?” **(T1D Patient 10)**  “If every person who was a diabetic, regardless of Type 1 or Type 2 had access to Google sensors that will send a reading to your phone, or your insulin pump, or whatever system you happen to be using, every 5 minutes – my thing sends a report to my insulin pump. And I know my blood sugar every moment of the day, and that’s been life changing for me. Testing your blood three times a day is not, or five times a day even, isn’t an effective way to manage diabetes. So if they could make it less expensive, or support people some way, to be able to have access to those, because it’s quite cost prohibitive for I think a lot of people.” (**T1D Patient 2**)  “So as a mental health professional, I have a client who actually has to choose between coming and getting trauma support with me or pay for her diabetic monitor. I mean, she can’t – she needs both, and she can’t afford both. And I’m sure there’s more people like her. So she’s choosing not to get her diabetic monitor, because she needs the trauma counseling right now.” (**T2D Patient 21**)  “it’s not that easy to get on for your health coverage – alternative health coverages. They won’t support a move to Ozempic or Jardiance, which is similar to Ozempic. So you either have pay out of pocket full price, or you stay on Metformin. And it’s not just one or two companies, because I thought, well, I’ll switch healthcare plans and the pharmacist says they’re all the same – you still have to get approved by whoever manages it and having hypoglycemia, and being stuck in a loop isn’t enough of a reason, even when you’re endocrinologist sends in saying that you need this prescription – they won’t approve it.” (**T2D Patient 21**) |
| **Access to Social and Mental Health Supports** | Many participants mentioned the link between mental health and managing their diabetes. Supports for mental health was emphasized as a priority, including mental health services and support groups for children and seniors.  Some patients also mentioned the need for supports when youth transition from pediatric to adult care | “hopefully should be more done about the mental health aspect of having chronic illness… you know just some advocacy there with employers or with schools, whatever it might be on handling the day to day grind of this, because it’s something every day you fix things…but it never really gets better. Never really goes away, right? You know this worked last time, might not work this week or whatever.” (**T1D Patient 7**)  “depression is like 70 per cent of the population, right? It’s not an oddity. There is something about the world we’re living in that isn’t working and for diabetics I think it needs to be proactive. I think it should be a built in part of all appointments, you know with the endocrinologist or something, maybe annually or something. There should be built in screening and referral if there is even a remote suspicion by any medical professional that this person might benefit. It’s just such a problem and that should also be funded, of course.” (**T1D Patient 13**)  “Support groups are really important. I was lucky. I lived in a bigger city. I was in Winnipeg. We had a youth diabetes group that I could go out to see. We went to camps. I learned a lot from other kids and from the people who were running these camps and groups and that education you know was far beyond what I got in the hospital. So I think having that opportunity is also important for the children.” (**T1D Patient 19**)  “sometimes you kind of get fed up, like my brain’s tired, I don’t want to have to carb count and calculate, right. I don’t want to have to inject, right? But you can’t really do that right – you can’t take a break from that right – if not your health kind of you know – you get in trouble for that, right. And then there’s the balance of like enjoying life, and enjoying all that and enjoying it, right?” (**T1D Patient 20**)  “you can also offer classes for adults so your parent have a better understanding of what’s going on. And then for you, it might be a little easier to see it in a playful way. Then your parents know exactly what to do, what to say, how to do it, how to count your sugars, how to count your dosages.” (**T1D Patient 24**)  “seniors support group. Yes, so because seniors, like my mom, I believe…she needs to talk to someone to share experiences. And she has only me to tell everything. And I may not feel the same way, I may not be compassionate enough all the time. But I believe if there will be a group of, there will be classes like that, or community support, groups like that, for seniors, together with a diabetes educator, who can have some sessions with them, and they can socialize, that would be definitely something that mom – I would take my mom there. And it will improve her psychological state related…to diabetes and side effects.” (**T2D Caregiver 2**)  “a support group would be really nice to be a part of, and to have. Because a lot of people don’t feel supported, and there’s a lot of things you can do in a support group, whether it be meal planning together or just sharing information.” (**T1D Patient 30**)  “if you think about newcomers and people who are priority populations, it's people that might not have access to diabetes care and prevention. And I think that we can't forget about those populations as well, because we're just going to overburden the system so that nobody is going to get the care that they deserve, because there's just going to be so much diabetes around us that we have to contend with… So I think they need to not forget about those priority populations.” (**T2D Caregiver 3)** |
| **Education- awareness for the public, patients, families, as well as clinicians** | Lack of education and awareness about diabetes (such as about the different types) among the public was mentioned as a concern by most participants. Some participants mentioned the need for diabetes awareness in schools. Some participants also mentioned the need for clinician education and awareness as well, especially about diabetes treatments and technologies. | “education on all parts for the people living with the disease. I learn something new every single day that I didn’t know. I’ve only had Type 1 for three years but every day I learn something new; every single day. And again the general public are unaware…or re-educating the public because I still think that there is that stigma that it’s you know people that are overweight and sedentary that get this disease and that’s not the case.” **(T1D Patient 1)**  “the lack of knowledge of diabetes among the community because the place I work, you know sometimes I will feel high, sometimes I feel low, which definitely impacts my productivity. Although I do – have given a full explanation to my employers that I am being a diabetic and this might impact my productivity sometimes. They might understand but they don’t fully comprehend the situation” **(T1D Patient 28)**  “Well, I wish somebody would be able to sit down with me and talk about my diet, because I just don't understand and it was many, many, many years ago that somebody talked to me about the diet, but I don't remember and it's confusing, and I wish I could just see a dietitian or somebody to help me figure this out.” (**T2D Caregiver 1**)  “I'd like to have more prevention initiatives in place. For example, I'm wondering if there are things that I'm doing that are putting me at risk (I don't have diabetes-- I'm 54). And for my children-- they are 13, 11, 10 years old. . . how can I make sure their risk is decreased?” (**T2D Caregiver 2**)  “But I haven't been to an education class for 10 years or so. And I think maybe that is something that people should be, if they are on diabetes medication, maybe they should be offered classes every year, every second year, just to call say, “*How are you doing? Would you like to come in and go to a class?*” I find that dieticians, when I did talk to dieticians years ago, it was – the options that they laid out, seemed to be extremely complicated.” (**T2D Patient 6**)  “senior centres…and having education seminars for the caregivers, so that we know what to look for. Honestly, when I go to see my mom, and she'll tell me, “*Oh, I'm dizzy*” and it's like, well, I think, is that her blood sugar, or is she just getting up too fast, or is that something else? What are the really concerning symptoms or signs that you might not be managing your diabetes through your exercise or diet, effectively? Or how do you recognise that? But I think people themselves need to be more partners in their care. So maybe pilot something, say, *“How about we try this and see how it works?”*” (**T2D Caregiver 2**)  “And I think in healthcare, we have to realise that it's spread across, everybody's accountable, it’s spread across a complete spectrum of health care, whether it be primary care or acute care. But I think we're responsible for our own health, but I also think that the system is responsible to ensure that we are educated about our health. And that's why I say accountability, it falls within primary care, but it also falls within the acute care side of it.” (**T2D Patient 3)**  “I wish maybe I had learned, not that diabetes is even generational or in our family, but if I had learned more about it, maybe in high school, during health class or something, I would have had more of an idea of the symptoms that I had had for years prior to actually getting diagnosed. And I never did get diagnosed with diabetes, until my liver actually failed, and they told me I had cirrhosis and I don’t drink. So I didn’t even know anything about what diabetes does to the body, until I was in a health crisis. So information that I learned in that program, in the LifestyleRx program, actually was so simple – it was like simple, stupid. They had this diagram of how the diabetic cycle works, and how it reinforces each other.” (**T2D Patient 21**)  “another huge barrier is that, for a lot of people, they actually don’t speak English…I think I heard somewhere that there’s over 120 languages just spoken in like the Calgary area. So if we have information available, it should be hopefully in like different languages to create awareness around diabetes, you know how to prevent it, manage it, meal plan, etcetera. Speaking from my own experience, like my grandma she doesn’t understand English, and sometimes we can go with her to appointments and sometimes we can’t, and it gets very difficult for someone who just doesn’t understand the language.” (**T2D Caregiver 12**) |
| **Access to Physicians and Specialists** | Some participants mentioned living in rural Alberta or outside of Edmonton and Calgary and not having access to certain specialists. Participants mentioned the need to have access to more specialists such as nurse educators, dieticians, and endocrinologists across Alberta. | “One thing that's lacking here in Central Zone is we don't have an endocrinologist in Red Deer. We don't have one at the hospital, we don't have one at the diabetic education centre. They consult with Edmonton and Calgary, but there isn't one here. So that is a huge hole in the system for Red Deer in Central Zone. So bringing those specialties into place, I think would really help to improve the care for adults living with diabetes and improve the peace of mind of the care partners for those people as well.” (**T2D Patient 5**)  “I think that the diabetic centres need to do a little bit more outreach and not wait for people to go in and contact…I was working very hard, travelling a lot and that sort of thing, I wasn't paying attention to my blood sugar at all. I went in for an A1C and I got a call from Alberta Health a couple of days later saying, “*We're just checking to see if you're still alive, because we saw what your A1C was. And we have registered you for a course, next Tuesday*”, and I said, “*I don't know if I could make it next Tuesday.*” And they said, “*You didn't hear me right. I've registered you for a course next Tuesday, you will be there*.” Now that was about 10 years ago.” (**T2D Patient 11**)  “ways our government can improve is improved access to primary care, just improving people's access to a family physician, to a nurse practitioner or to a diabetes educator.” **(T2D Patient 4)**  “I would recommend you to ask your family doctor to get referred to endocrinologist, because what my endocrinologist also does, she checks my feet, just to make sure that they don’t have neuropathy and things like that. And she checks my hormones, which is very important. This is the thing that family doctor may miss, but the endocrinologist will always check it every time I come in.” (**T1D Patient 24**)  “There’s lots of gaps in our healthcare for both type 1 and type 2. Type 1 you are very well supported through the Children’s Hospital. But even that is getting worse because the amount of people that are newly diagnosed continues to increase, and so the nurse to patient ratio continues to increase. So there’s not as close monitoring as there used to be, or connectedness to your nurse at the hospital as there used to be.”(**T1D Caregiver 25**)  “just the support in the school systems. There is no school nurses anymore, really, because there was no funding for that, so it lands on the TAs, which again, are not medically trained at all.” (**T1D Caregiver 25**)  “most of the time you’re right, children get Type 1 diabetes. So they have access to insulin pumps and access to almost every child who – I’m sure who is diagnosed has access to the programs through the major health centers. But those are only in Edmonton, and those five centers that do the pumps anyways are only in Edmonton, Calgary and Grand Prairie. So if you don’t live in one of the major hubs, you have a lot of hours’ worth of traveling and struggles to be educated and have the top-notch coverage. And that shouldn’t be – that disparity shouldn’t happen in our province.” (**T1D Patient 2**) |
| **Compassionate care and flexibility in healthcare** | Participants spoke about not feeling listened to and taken seriously when they tried to express their concerns about their diabetes management with their physicians. Participants expressed the need for their care teams to be open-minded, compassionate, and listen to the needs of patients. | “everyone’s body is different so they give you all these blanket rules like – you know in terms of like treating your lows and like how long you have to wait and everything like that. But I found that there is a bit of a need for flexibility because everyone’s body reacts differently. I kind of tried to discuss that with them and say like, *“Hey, actually, this isn’t working for me so I’m doing this”* and then they were like, *“No, no, no, you have to do this.”*” (**T1D Patient 6**)  “The negative stereotype of becoming a Type 2 diabetic is extremely – it’s a big barrier. People say it’s because you’re a fat lazy person who eats the wrong things, and so the treatment you get from say the pharmacy, the pharmacists, people in general, the people around you, is, in my experience has been fairly negative and usually very blaming. And when you try to explain them that that’s not kind of how it works, it doesn’t get received well.” **(T2D Patient 18)**  “a patient always tells stories and they tell their story based on their personal experience. And I think what we don't recognise in the system is the patient telling a story to their doctor, it's a selfless act…And I think that they're revisiting their life experience, they're really visiting their trauma, but just to get their story across to the doctor. And the whole objective behind them is they want to improve the future of other patient’s outcome.” (**T2D Patient 25)**  “For her it's like, *oh, the doctor knows best*. *And just don't ask too many questions, don't be too much of a pain*. But I think if it was setup so that in those appointments with whoever it is, that she was being listened to, and respected.  And there was this working together kind of vibe, I think that would be really helpful for my mom. And I think she would just kind of find those small wins and bring that home, and then talking to me about it” **(T2D Caregiver 2)**  “I've had to tell my own physician, “*I have more questions. Please sit still for five minutes while we go through this.”* Because they're often in, they're just in a hurry, because they have so much other pressures and things going on with their clinics. But I think sometimes patients forget that they can ask for more time with their physician, and you certainly have that option to ask for longer appointment times.” (**T2D Patient 7**)  “We need to have professionals who are open-minded…and ask us what do we need and how can we help you, and let’s work with where you are right now. Because you can tell me you need to eat this this and this, and I’m going to be like but I don’t like that, that or that. But I’ll eat this, this and this, and how can we work that into my lifestyle. So I think that open-mindedness is huge for our treatment.” (**T1D Patient 10**)  “there are many instances I've had, where the diets they suggest (in my case demand) you to take is very unrealistic as we are all very different and have very different needs when it comes to dieting and what we can or can't handle/are willing to do. Because unlike a weight loss diet this is a lifelong change that needs to be maintained and it's very challenging when we’re told we have to change the way we eat and how we think about food. I think doctors need to keep this in mind because it's very easy to develop eating disorders or other types of mental health illnesses when we are constantly told that '"your diet isn't good enough, your A1C isn't good enough" and being shamed for having one cheat day or consuming something that doesn't follow their diet plan they make you follow.” (**T1D Patient 11**) |
| **Access to physical activity** | Affordability of spaces for physical activity | “I would say improving people's access to exercise, people's access to getting more active and lowering the cost of those things. So getting access to getting into the pool for people that have low mobility, getting access to indoor walking tracks, especially for seniors with lower incomes, would really make a big difference.” (**T2D Patient 9**)  “you can just give me information, need to exercise every day, but we are a winter country. We are in a winter country, you provide money to people so that they can exercise every day… So, there needs to be more initiative in different communities, helping people to make sure that the prevention is clear” (**T2D Caregiver 10**)  “Exercise opportunities – up here in the north, once it gets to minus 40, that’s your going outside is limited, if you don’t have the right equipment. Schools are limited in the exercise opportunities they offer kids. And even in the summer, there’s not a lot to support that. It would be nice to see more support for any getting out, getting movement” **(T2D Patient 18)** |
| **Improved patient and community informed research capacity** | Participants wanted ongoing and continued engagement of patient and families in care and in research | “maybe do a survey of the diabetic community and see what they want. This focus group is really helpful and maybe more of this.” (**T2D Caregiver 23**)  “connecting academics to community members, those who are living with diabetes, and the people that care for them. So I think doing more community based research, so what's needed and what's working, where are the gaps. And I think that maybe some of the gaps – [University of Alberta Researcher] has done a lot of work around like First Nations communities, there's increasing prevalence of diabetes in that population.” **(T2D Caregiver 24)** |
| **Building awareness of diabetes research** |  | “I think that the Edmonton Protocol is great that people are willing to give their money to diabetes and make donations, but this is ridiculous. This should be funded – and funded substantially. Because we, as Type 1s and Type 2s, if we do not manage our blood sugars, we’re going to cost the system a whole lot of money. And we are going to suffer, and our families are going to suffer, and our children are going to suffer, and it’s not acceptable.” (**T1D Patient 26**)  “I think that research and development should be also focusing on when it comes to diabetes care and prevention on side effects and side conditions. When we talk about diabetes, although people feel isolated, the disease is not isolated, especially in the type 2 diabetes it’s part of metabolic syndrome. It involves kidneys, it involves neuropathy…B12 depletion, if people are on metformin for a long time. The eyes, vision. Eventually it all complicated, the entire body that fails, right? So it would be nice to have more research and development on looking at diabetes as a part of the overall body condition, considering the side effects and a parallel condition, conditions that develop in parallel.” (**T2D Caregiver 2**)  “I really don't know what research is going on. But I believe around the world, there are some very, very bright people who are spending an awful lot of time and attention to it. I just don't know.” (**T2D Patient 22**)  “making the information available to people in plain language. So if they get partnerships with academics or communities, just kind of make it so that it's understandable in plain language, so that if English isn't your first language, or if you're elderly and maybe you have problems with memory, it might really come down to like what was said, just don't eat anything white. It might get real basic like that.” (**T2D Caregiver 20**) |
