# Supplementary material for: Living With Diabetes in Alberta: Patient and Caregiver Priorities for Diabetes Care, Management, and Treatment
Source: Health Expect. 2026 Feb 5;29(1):e70587. doi: 10.1111/hex.70587 (PMC12877417; doi:10.1111/hex.70587)
Supplement: Supplementary file 2 — coding tree. [file HEX-29-e70587-s002.docx]

Appendix. Coding Tree of the Analysis
